# Bidirectional causality between the levels of blood lipids and endometriosis: a two-sample mendelian randomization study

**DOI:** 10.1186/s12905-024-03213-w

**Published:** 2024-07-04

**Authors:** Zhenna Wang, Chunxian Zhan, Linghua Liao, Ye Luo, Shunhe Lin, Shihan Yan

**Affiliations:** Department of Gynaecology and Obstetrics , Fujian Maternity and Child Health Hospital, College of Clinical Medicine for Obstetrics & Gynecology and Pediatrics, Fujian Medical University, No.18, Daoshan Road, Gulou District, Fuzhou City, Fujian Province China

**Keywords:** Blood lipid, Endometriosis, Genome-wide association study, Mendelian, Randomization, The causal effect and single nucleotide polymorphism

## Abstract

**Background:**

Observational studies have found a correlation between the levels of blood lipids and the development and progression of endometriosis (EM). However, the causality and direction of this correlation is unclear. This study aimed to examine the bidirectional connection between lipid profiles and the risk of EM using publicly available genome-wide association study (GWAS) summary statistics.

**Methods:**

Eligible exposure variables such as levels of triglycerides (TG), total cholesterol (TC), low-density lipoprotein (LDL), and high-density lipoprotein (HDL) were selected using a two-sample Mendelian randomization (MR) analysis method following a series of quality control procedures. Data on EM were obtained from the publicly available Finnish database of European patients. Inverse variance weighted (IVW), MR Egger, weighted median, and weighted mode methods were used to analyze the causal relationship between lipid exposure and EM, exclude confounders, perform sensitivity analyses, and assess the stability of the results. Reverse MR analyses were performed with EM as exposure and lipid results as study outcomes.

**Results:**

IVW analysis results identified HDL as a protective factor for EM, while TG was shown to be a risk factor for EM. Subgroup analyses based on the site of the EM lesion identified HDL as a protective factor for EM of the uterus, while TG was identified a risk factor for the EM of the fallopian tube, ovary, and pelvic peritoneum. Reverse analysis did not reveal any effect of EM on the levels of lipids.

**Conclusion:**

Blood lipids, such as HDL and TG, may play an important role in the development and progression of EM. However, EM does not lead to dyslipidemia.

**Supplementary Information:**

The online version contains supplementary material available at 10.1186/s12905-024-03213-w.

## Introduction

Endometriosis (EM) is a chronic condition that is characterized by the presence of ectopic endometrial tissue outside of the uterine cavity, ectopic endometrial tissue cyclic bleeding, fibrosis [1]. The disease affects 6-10% of the fertile female population and seriously impacts the reproductive health and quality of life of women [2].

Typical symptoms of EM include dysmenorrhea, chronic pelvic pain, menstrual abnormalities, and even infertility. Studies also show that over 40% of women with EM present with central sensitization (CS) [3] that alters pain perception, exacerbates pain symptoms, predisposes women with EM to the development of other chronic conditions, and could lead to worse response to treatments [4].

Pathophysiology of EM is complex and involves chronic inflammation, hormonal changes, genetic and epigenetic factors, altered metabolism, local immune dysregulation [5]. Current studies suggest that abnormal lipid metabolism may also contribute to the development, severity and progression of EM. A prospective observational cohort study that pooled data of 1,299,349 females with up to 20 years of follow-up, found that women with EM had lower body mass index and a peripheral body fat distribution (waist-to-hip ratios below 0.60) [6]. This suggests that abnormalities in lipid metabolism that are manifested by changes in the lipid levels in the peripheral blood [7] may be related to the progression of EM. A retrospective study also found a positive correlation between the levels of TG and the severity of EM [8]. Furthermore, the risk of atherosclerosis was higher in the population of patients with EM, possibly due to long-term chronic inflammation that exacerbates this process [9]. These findings seem to suggest that dyslipidemia is an adverse outcome of the progression of EM. Additionally, dyslipidemia may affect the efficacy of EM treatment. In the rat EM model, antilipidemic treatment decreased the size of endometrial lesions [10]. By using a mouse model, Heard, ME et al. [11] demonstrated that increased fat intake significant affected the size of EM lesions. Studies also showed that dyslipidemia persisted after the pharmacological treatment of EM which was effective in controlling the symptoms or shrinking the lesions [12, 13]. Therefore, understanding the role of lipids in the pathophysiology of EM, and the causal relationship between the two may provide a theoretical basis for the long-term management of EM by adjusting the dietary structure or the use of lipid-lowering drugs.

Mendelian randomization (MR) uses specific single nucleotide polymorphisms (SNPs) as instrumental variables to identify potential causal associations between exposures and outcomes [14]. MR can be considered a natural randomized clinical trial based on the genetic law of “random assignment of parental alleles to offspring”, which allows to reliably infer causality by avoiding potential confounders or reverse causality in prospective or retrospective observational studies.

In this study, we used a two-way MR approach to investigate whether there is a causal relationship between the levels of triglycerides (TG), total cholesterol (TC), low-density lipoprotein (LDL), high-density lipoprotein (HDL) and EM.

## Methods

### Objective

To explore the bidirectional causal associations between blood levels of the lipid quartet (HDL, TG, TC, and LDL) and EM by two-way MR analysis using GWAS data.

### Data source

Data on blood lipid levels were obtained from the Global Lipid Consortium database [15, 16], which included a total population of 1,654,960 patients. EM data were obtained from the Finnish database version R.9 (ICD-10: N14), with a sample size of 15,088, a control group of 107,564, and a number of SNPs of 20,141,087 (https://r9.risteys.finngen.fi/endpoints/N14_ENDOMETRIOSIS). Analysis of EM subtypes at different sites was performed. To avoid bias caused by confounding factors such as ethnicity, we selected only the genetic background of people with European ancestry for the analysis.

The composition of the population included in the analyses for the different sites was as follows: deep EM (*n* = 2856), EM of the fallopian tube (*n* = 213), EM of intestine (*n* = 436), unspecified EM (*n* = 2982), EM of ovary (*n* = 5867), EM of pelvic peritoneum (*n* = 5628), EM of rectovaginal septum and vagina (*n* = 2456), and EM of uterus (*n* = 4267).

**Fig. 1:**
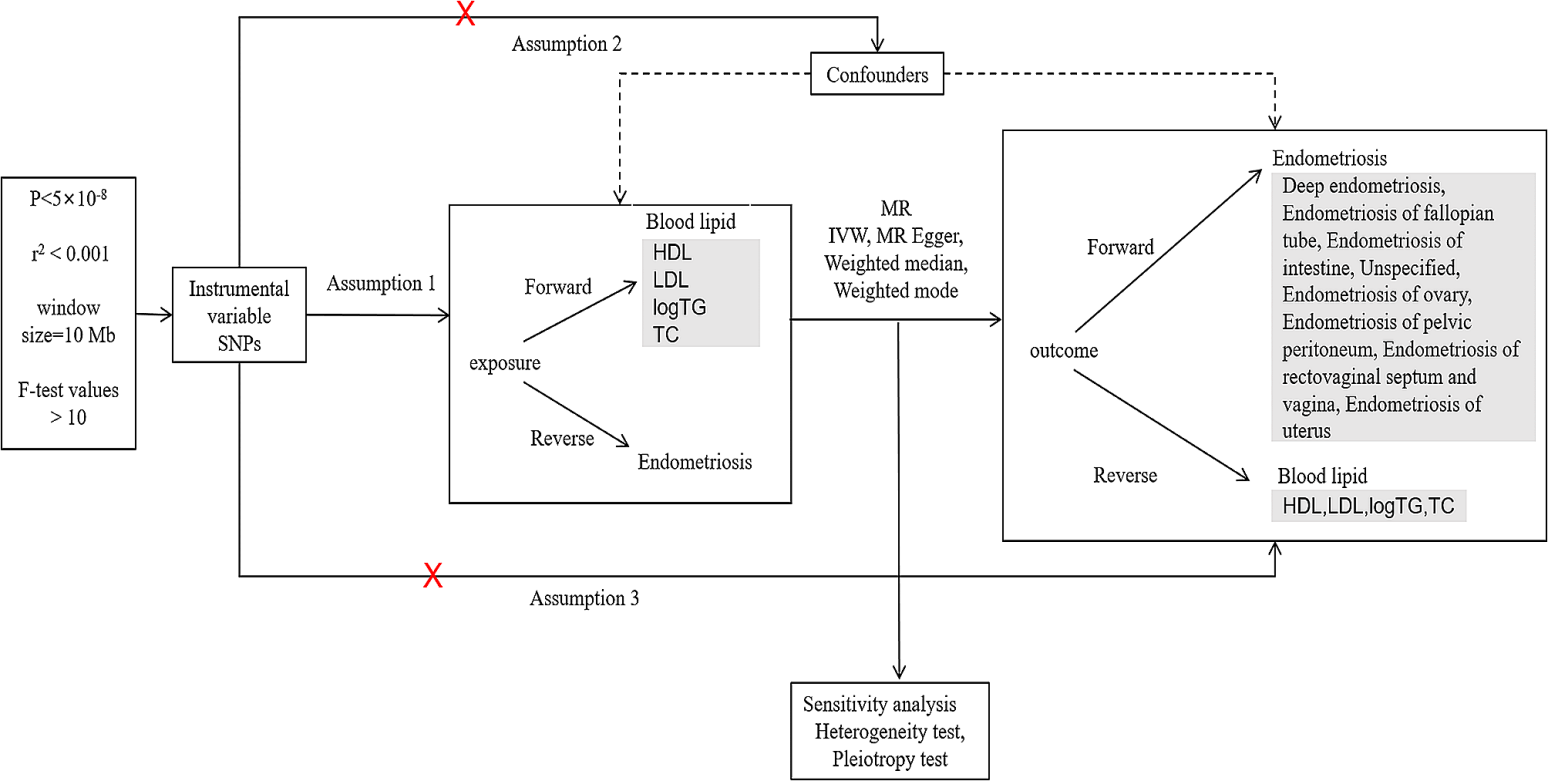
Flowchart of the MR analysis

A flowchart of the study design is summarized in Fig. 1.

Since the data source that was used in this study obtained informed consent from all participants, separate institutional review board approval was not required.

### Instrumental variable

Screening criteria were as follows: (i) the instrumental variable strongly correlated with the exposure factor, (ii) confounders associated with the outcome were excluded, and (iii) the outcome could be influenced only by the exposure and not by the instrumental variable itself [17, 18]. Conditions required for significant correlation of the instrumental genetic variant were as follows: *p* < 5 × 10 − 8, r2 < 0.001, genetic distance = 10 000 kb, and all F-test values > 10. Using the website phenoscanner database (http://www.phenoscanner.medschl.cam.ac.uk) [19, 20], all phenotypes associated with the instrumental variables were searched, and SNPs associated with outcome and confounders (*P* < 5 × 10 − 8) were excluded from the instrumental variables for the multiplicity of validity. The confounders of EM were based on the results of the reference article [21]. For forward univariate MR analyses, exposure factors were as follows: HDL, TG (logarithmic), TC, and LDL. EM was selected as an outcome of interest. Reverse MR analyses were performed with EM set as an exposure factor and the lipid levels as the outcome. Setup parameters were equivalent to those of the forward univariate MR analyses.

### Statistic analysis

Univariate MR analysis used inverse variance weighted (IVW) to calculate odds ratio (OR) and 95% confidence interval (CI), and MR Egger, weighted median, and weighted mode [22] were used as supplementary methods to verify the stability of the results. Sensitivity analyses included a heterogeneity test and horizontal multiplicity test to assess the presence of potential bias in the results. *P* < 0.05 in Cochran’s Q-test indicated the presence of heterogeneity [23]. Multiplicity of SNPs was measured by MR Egger regression, and the intercept term *p* < 0.05 indicated the presence of horizontal multiplicity in the results [24]. “Leave-one-out” gradually eliminated each SNP, calculated the meta-effects of the remaining SNPs, and observed whether the results changed after the elimination of each SNP. When the results matched the total effect size of the MR analysis, the analysis was considered robust [14].

Two Sample MR packages (version 0.5. 6), and Radial MR package (version 1.0) in R software (4.2.2) were used for analysis, with a test level of α = 0.05.

## Results

### Positive mendelian analysis of lipid quartiles and EM

#### Instrumental variables for screening

**Fig. 2:**
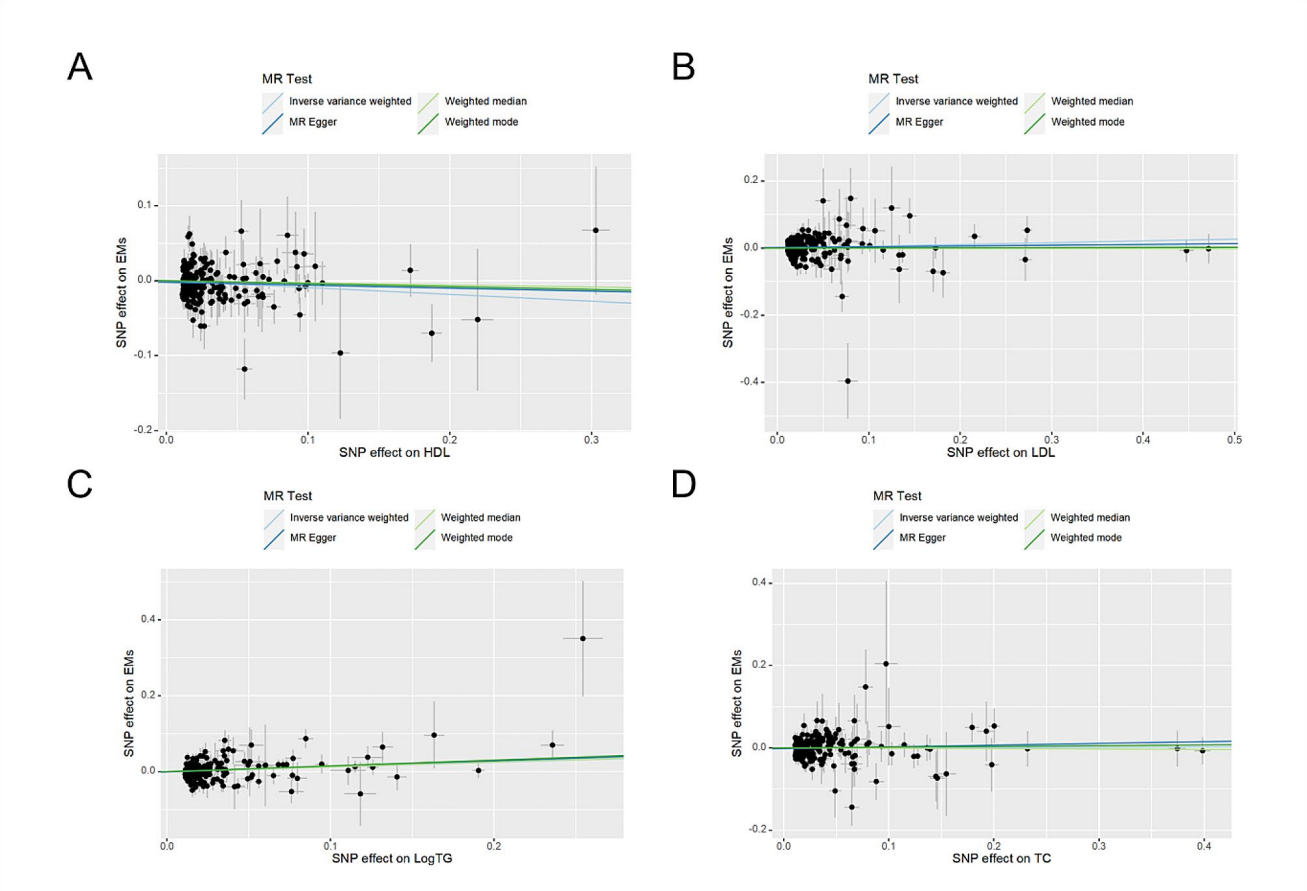
Scatter plot showing the causality of four blood lipid on EM identified by IVW, MR egger, weighted median and weighted mode; (**A**) HDL; (**B**) LDL; (**C**) Log TG; (**D**) TC

A total of 323 SNP_HDL_, 295 SNP_logTG_, 315 SNP_TC_, and 279 SNP_LDL_ were obtained after the removal of linkage disequilibrium (*P* < 10^− 8^). Of them, 256 SNP_HDL_, 241 SNP_logTG_, 249 SNP_TC_, and 235 SNP_LD_, were strongly associated with EM after removing the unavailability of matches or palindromes. Finally, 206 SNP_HDL_, 189 SNP_logTG_, 221 SNP_TC_, and 210 SNP_LDL_ were included in the MR analyses after removing confounders associated with EM. The F statistics were greater than 10, indicating that the selected SNPs did not have weak instrumental variable bias. We used IVW, MR Egger, weighted median, and weighted mode to assess the causal relationship between the lipid quartet and EM (Table 1). Scatter plots showed that the analyses had consistency (Fig. 2).

**Table 1:** MR results

Blood lipid	SNPs	Method	β	SE	OR95%(CI)	*P*
HDL	206	IVW (stochastic effect)	-0.091	0.041	0.913(0.843–0.989)	0.025
	206	MR Egger	-0.039	0.066	0.961(0.845–1.093)	0.548
	206	Weighted median	-0.028	0.056	0.972(0.871–1.086)	0.620
	206	Weighted mode	-0.039	0.057	0.962(0.860–1.086)	0.497
logTG	189	IVW (stochastic effect)	0.123	0.042	1.131(1.041–1.228)	0.004
	189	MR Egger	0.145	0.063	1.156(1.022–1.308)	0.022
	189	Weighted median	0.126	0.062	1.134(1.005–1.280)	0.041
	189	Weighted mode	0.152	0.051	1.164(1.052–1.287)	0.004
TC	221	IVW (stochastic effect)	0.004	0.035	1.004(0.937–1.076)	0.900
	221	MR Egger	0.042	0.050	1.043(0.945–1.151)	0.406
	221	Weighted median	-0.009	0.058	0.991(0.885–1.109)	0.871
	221	Weighted mode	0.018	0.048	1.018(0.926–1.118)	0.717
LDL	210	IVW (stochastic effect)	0.054	0.037	1.055(0.982–1.134)	0.144
	210	MR Egger	0.023	0.050	1.023(0.927–1.129)	0.650
	210	Weighted median	-0.004	0.053	0.996(0.897–1.105)	0.936
	210	Weighted mode	0.005	0.043	1.005(0.924–1.093)	0.913

MR, mendelian randomization; IVW, inverse variance weighted; SE, standard error

As shown in Table 1, the inverse variance weighted (IVW) model identified HDL as a protective factor for EM, with OR = 0.913, CI = 0.843–0.989, *P* = 0.025. On the other hand, logTG was a risk factor for EM (OR = 1.156, CI = 1.022–1.308, *P* = 0.022). TC and LDL had no significant causal relationship with EM (*P* > 0.1 for all four model results).

#### Sensitivity analysis

To test for the presence of bias in the MR analysis, further sensitivity analyses were performed. Cochran Q test results revealed heterogeneity in SNPs (*P* < 0.001). Therefore, we next focused on the results of the IVW random effects model (Table 2).

**Table 2:** Cochran Q test results: Heterogeneity test

	MR egger			IVW		
	Q	df	*P*	Q	df	*P*
HDL	281.472	204	< 0.001	282.867	205	< 0.001
logTG	261.823	187	< 0.001	262.144	188	< 0.001
TC	280.629	219	< 0.001	282.021	220	< 0.001
LDL	303.743	208	< 0.001	304.903	209	< 0.001

MR, mendelian randomization; IVW, inverse variance weithted; Q, Quantifier; df, degree of freedom

**Fig. 3:**
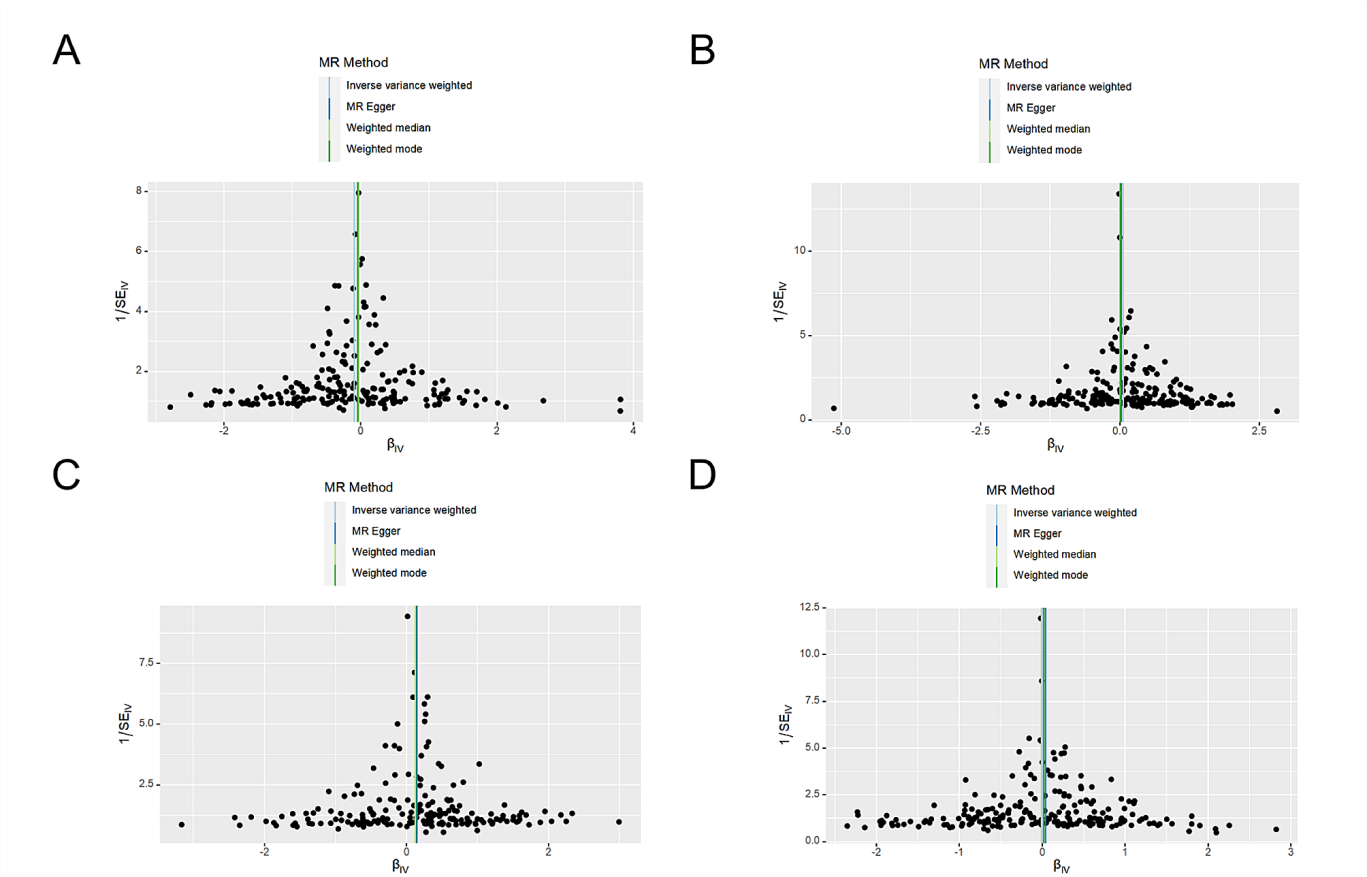
Funnel plots showing the causality of four blood lipid on EM identified by IVW, MR egger, weighted median and weighted mode; (**A**) HDL; (**B**) LDL; (**C**) Log TG; (**D**) TC

The results of the funnel plot (Fig. 3) showed a largely symmetrical distribution of SNPs included in the analyses, suggesting that the inferred causal effect was less affected. As shown in Table 3, the pleiotropy test showed no horizontal multiplicity of SNPs (*P* > 0.05). In addition, we used the leave-one-out method to confirm the effect of HDL, logTG, TC, and LDL on EM potential outliers in the instrumental variables for causal effects. The removal of any individual SNP did not have a large impact on the results (Supplementary Figs. 1–4), indicating that the results of the MR analysis were robust and reliable.

**Table 3:** Egger intercept test results: Pleiotropy test

MR egger	Intercept	SE	*P*
HDL	-0.002	0.002	0.316
logTG	-0.001	0.002	0.632
TC	-0.002	0.002	0.298
LDL	0.002	0.002	0.374

MR, mendelian randomization; SE, standard error

### Inverse mendelian analysis of blood lipid profile and EM

**Fig. 4:**
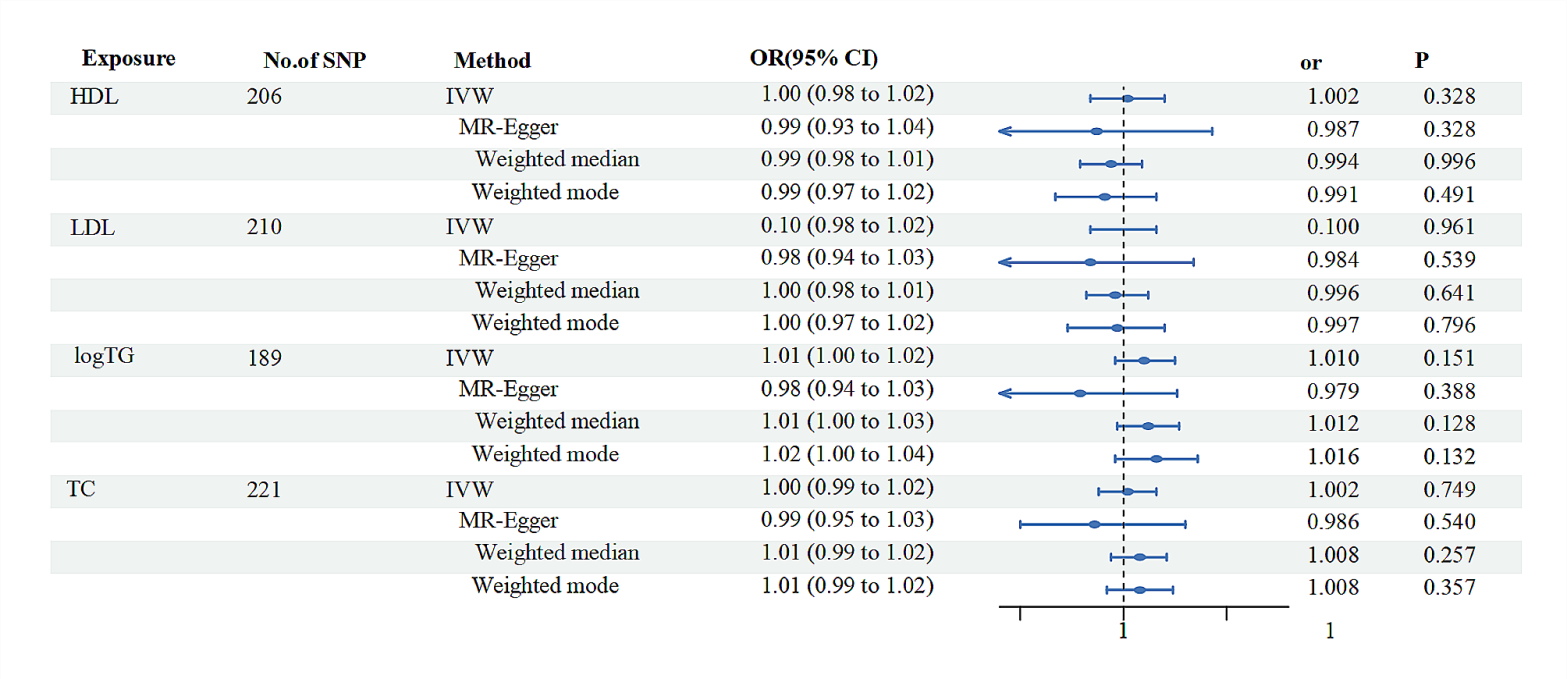
The results of four different methods of reverse MR analysis

A total of 27 SNPs with a genome-wide threshold of significance (*P* < 5 × 10^− 8^), associated with EM, were identified and included in the analyses after removing sequences that could not be matched to lipids or had palindromic sequences (21 SNP_HDL_, 19 SNP_logTG_, 22 SNP_TC_, 22 SNP_LDL_. No significant evidence of a causal effect of EM on lipids was found on IVW, weighted median and weighted mode analyses (Fig. 4, Supplementary 5). The results of the multiple validity test showed that the intercepts of the MR Egger regressions were 0.002 (HDL, LDL, TC) and 0.004 (logTG), respectively, with P_HDL_ = 0.567, P_HDL_ = 0.527, P_HDL_ = 0.179, and P_HDL_ = 0.451, suggesting that there was no potential horizontal versatility. Leave-one-out method showed similar results (Supplementary Fig. 6).

### Subgroup analyses

Subgroup analyses were then performed based on the site of endometriosis lesions. Exposure factors included HDL, LDL, logTG, and TC, and the following outcomes were selected: deep EM, EM of fallopian tube, intestine, unspecified EM, EM of ovary, EM of pelvic peritoneum, rectovaginal septum and vagina, and uterus.

As shown in Table 4, HDL was identified as a protective factor for EM of uterus, IVW (OR = 0.837, CI = 0.731–0.959, *P* = 0.01), while logTG was a risk factor for the EM of fallopian tube (OR = 1.946, CI = 1.093–3.464, *P* = 0.02), EM of ovary (OR = 1.149, CI = 1.027–1.286, *P* = 0.02), and EM of pelvic peritoneum (OR = 1.186, CI = 1.035–1.360, *P* = 0.01).

**Table 4:** MR results of four blood lipid based on location of lesions by inverse variance weighted methods

Blood lipid	Method	location of lesions	SNPs	β	SE	OR95%(CI)	*P*
HDL	IVW	Deep endometriosis	207	0.023	0.077	1.023(0.879–1.190)	0.77
		Endometriosis of fallopian tube	206	-0.353	0.281	0.703(0.405–1.218)	0.21
		Endometriosis of intestine	206	0.277	0.189	1.320(0.911–1.911)	0.14
		Unspecified	205	-0.122	0.082	0.885(0.754–1.039)	0.14
		Endometriosis of ovary	205	-0.089	0.058	0.915(0.816–1.025)	0.13
		Endometriosis of pelvic peritoneum	204	-0.050	0.065	0.951(0.837–1.081)	0.45
		Endometriosis of rectovaginal septum and vagina	207	-0.024	0.087	0.976(0.823–1.158)	0.78
		Endometriosis of uterus	206	-0.178	0.069	0.837(0.731–0.959)	0.01
LDL	IVW	Deep endometriosis	210	0.081	0.069	1.085(0.948–1.241)	0.23
		Endometriosis of fallopian tube	210	0.061	0.237	1.062(0.667–1.691)	0.80
		Endometriosis of intestine	210	0.233	0.166	1.262(0.912–1.747)	0.16
		Unspecified	210	0.092	0.065	1.096(0.966–1.244)	0.16
		Endometriosis of ovary	210	0.039	0.055	1.040(0.933–1.160)	0.48
		Endometriosis of pelvic peritoneum	206	0.077	0.050	1.080(0.979–1.191)	0.12
		Endometriosis of rectovaginal septum and vagina	210	0.044	0.077	1.045(0.898–1.216)	0.57
		Endometriosis of uterus	209	0.074	0.059	1.077(0.959–1.209)	0.21
logTG	IVW	Deep endometriosis	189	0.007	0.087	1.007(0.850–1.194)	0.93
		Endometriosis of fallopian tube	189	0.666	0.294	1.946(1.093–3.464)	0.02
		Endometriosis of intestine	189	-0.387	0.202	0.679(0.457–1.010)	0.06
		Unspecified	187	0.093	0.082	1.098(0.934–1.290)	0.26
		Endometriosis of ovary	189	0.139	0.057	1.149(1.027–1.286)	0.02
		Endometriosis of pelvic peritoneum	187	0.171	0.070	1.186(1.035–1.360)	0.01
		Endometriosis of rectovaginal septum and vagina	189	0.067	0.094	1.069(0.890–1.284)	0.48
		Endometriosis of uterus	189	0.052	0.074	1.053(0.911–1.217)	0.48
TC	IVW	Deep endometriosis	221	0.033	0.071	1.034(0.899–1.189)	0.64
		Endometriosis of fallopian tube	221	0.169	0.243	1.185(0.736–1.907)	0.49
		Endometriosis of intestine	221	0.283	0.170	1.327(0.951–1.852)	0.10
		Unspecified	220	0.040	0.070	1.041(0.908–1.193)	0.56
		Endometriosis of ovary	221	-0.005	0.058	0.995(0.888–1.115)	0.93
		Endometriosis of pelvic peritoneum	218	0.060	0.055	1.061(0.954–1.181)	0.27
		Endometriosis of rectovaginal septum and vagina	221	-0.006	0.078	0.994(0.854–1.157)	0.94
		Endometriosis of uterus	220	-0.008	0.058	0.992(0.886–1.112)	0.89

MR, mendelian randomization; IVW, inverse variance weithted; SE, standard error

LDL and TC had no genetic factor effects on the various subtypes of EM. There was no evidence of horizontal pleiotropy of SNPs (*p* > 0.05).

MR results of four blood lipids based on the location of lesions, and Egger intercept test results are shown in Supplementary Tables 1 and 2.

## Discussion

In this study, we explored the bidirectional causal associations between blood levels of the lipid quartet (HDL, TG, TC, and LDL) and EM by two-way MR analysis using GWAS data to provide a theoretical basis for the adjustment of lipid metabolism and dietary interventions in the long-term management of EM.

Bidirectional MR analyses showed that HDL reduced the risk of developing EM, whereas TG was a risk factor for EM. On the other hand, EM did not affect the values of HDL, TG, TC, and LDL. Our results suggest that HDL and TG may play a key role in the pathophysiological process of EM. Subgroup analyses based on the site of the EM lesion identified HDL as a protective factor for endometriosis of the uterus, while TG was identified as a risk factor for the EM of the fallopian tube, ovary, and pelvic peritoneum. Several previous cross-sectional studies have shown increased levels of lipid metabolites in the blood of women suffering from EM [25–28]. In a prospective cohort study, Naoko Sasamoto et al. found that disorders of lipid metabolism were strongly associated with chronic pelvic pain due to EM by comparing lipid metabolites of preoperative and postoperative EM patients [29]. Another prospective cohort study reported that women with EM had a higher risk of hypercholesterolemia and hypertension compared to women without the condition [30]. Moreover, EM patients had higher arterial stiffness [9] and incidence of cerebrovascular-related headaches [31]. In addition, Poeta do Couto, C et al. [32] found that patients diagnosed with EM had a significantly higher risk of cardiovascular disease. Considering the complexity of lipid metabolism and EM, the causal relationship between the two has not been completely clarified. Our results suggest that clinicians should pay special attention to the HDL and TG profiles of patients in the long-term management of EM, which may help to control or delay the progression of this disease.

Numerous studies showed that the lesions of EM patients are caused by ectopic endothelial cell implantation outside the uterine cavity and that the local inflammatory response promotes angiogenesis, cyclic bleeding, hemostasis, and accelerated tissue fibrosis formation [33]. Angiogenesis, cyclic hemorrhage, and local inflammatory response are all important parts of the disease progression in EM. Coagulation plays a key role in the inflammatory response and angiogenesis. Li, Yan, et al. used MR to explore the causal association between coagulation factors and EM and found that the cascade of local coagulation and anticoagulation mediated by coagulation factors is an important cause for the development of EM. They also showed that the local aggregation of platelets triggered by the Von Willebrand factor (vWF) is a protective factor against EM [34]. vWF, which can serve as a reflection of the degree of vascular endothelial cell damage, is mainly synthesized by vascular endothelial cells, and is an indispensable bridging factor in the process of inducing platelet adhesion and aggregation [35]. Previous studies have demonstrated that HDL has anti-vascular endothelial oxidation, inflammation, and platelet aggregation functions [36]. HDL ameliorates and repairs endothelial cell damage by decreasing the level of inflammatory response and inhibiting LDL oxidation [37]. In addition, low levels of HDL and impaired vascular endothelial function result in elevated vWF levels in peripheral blood [38]. We hypothesize that HDL reduces the development of EM possibly by stimulating or working in synergy with vWF. Reduction of vascular proliferation and inhibition of ectopic lesion formation by HDL is one of the potential pathways for its role as a protective factor against EM.

Studies show that HDL reduces peripheral blood cholesterol levels by reverse transporting cholesterol to the liver and metabolizing it [39]. Various dietary and environmental factors can influence blood cholesterol levels [40]. For instance, dietary cholesterol increases serum total cholesterol and HDL [41]. Cholesterol in peripheral blood is an important source of steroid hormone synthesis in the body, and same abnormalities of cholesterol metabolism are present in EM patients [30], suggesting an potential role of cholesterol synthesis regulation in EM. We, therefore, may hypothesize that lowering estrogen synthesis by elevating peripheral blood levels of HDL through dietary management may delay EM progression. Previous studies have found high levels of aromatase and estrogen receptors in ectopic lesions [42]. High estrogen was shown to increase the release of cytokines and chemokines from macrophages, exacerbating the local inflammatory response and promoting the growth and invasion of ectopic lesions [43]. Estrogen precursor, a homeobox protein HOXA10, is highly expressed in endometrial mesenchymal stromal cells and acts through the steroid hormone-cholesterol synthesis pathway [44]. Levels of HOXA10 positively correlate with HDL and negatively correlate with TG in peripheral blood [45]. Cirillo, M et al. [46] found that the Mediterranean Diet lowered peripheral blood cholesterol and improved metabolic and oxidative status, as well as improved overall quality of life of EM patients. Therefore, modifying HDL through dietary modification to slow down the progression of EM is a feasible future direction.

Studies by Crook, D [47] and Melo, AS [48] found higher TG levels in EM patients. The results of these studies showed that patients with EM stages I-II had significantly higher levels of TG [49], while patients in stages III-IV showed a similar trend with worse lipid profiles and significant correlation with c-reactive (CRP) levels [48], suggesting that TG may be exacerbating the course of EM through the inflammatory response pathway. A cross-sectional study Li, Baijia et al. [7] found a high rate of metabolic syndrome in EM patients and associated it with high levels of peripheral blood TG which is involved in the atherosclerotic process, and increases the risk of cardiovascular disease [9] and metabolic syndrome [7].

In contrast to previous observational studies, our study accounted for confounding factors to a greater extent, provided bidirectional causal associations, and identified key risk and protective factors for EM from a genetic perspective. Our study may have clinical implications that may change the approach to the diagnosis and treatment of EM. Combined assessment of HDL and TG lipid profiles may be potentially used for screening patients and establishing early diagnosis and staging of EM. This, in turn, may contribute to a more multimodal approach to the treatment, since abnormal lipid profiles are significant risk factors of systemic comorbidities such as cardiovascular diseases [50]. Moreover, oral contraceptives are often used in the treatment of EM, as they are considered safe and efficient in the reduction of ovarian endometrioma size [51, 52]. However, the use of oral contraceptives was reported to be associated with significantly higher concentrations of high-density-lipoprotein cholesterol, and increased incidence of stroke and myocardial infarction [53]. The results of this study further emphasize the importance of a more informed approach to contraceptive prescription in EM patients due to potentially lipid higher levels in this population of patients. In addition, our findings provide a potential therapeutic approach to management and prevention of progression and postoperative recurrence of EM through modulating HDL and TG levels by diet or medication.

There are some limitations of this study. The results of the MR analysis were based on the European population, introducing a potential ethnic bias and limiting the extrapolation of causality. SNPs that were used for the analysis may correlate with other traits due to genetic polymorphisms, creating a confounding bias that may have impacted the accuracy of the causal inference. Our data were not stratified according to the different stages of the EM, and age, which may have resulted in some bias. We also acknowledge that further clinical studies are needed to investigate the effect of modifying lipid profiles of patients on endometriosis. Since this study was based on the data that were obtained from publicly available databases, we were unable to address these points in the scope of this paper. Further studies are needed to perform independent validation of our results.

## Conclusion

Bidirectional MR analysis found that hereditary HDL and TG levels were closely associated with the risk of developing EM. Our results suggest the need to focus on lipid levels in the long-term management of EM. Adjustment of dietary structure or use of lipid-lowering drugs instead of hormonal therapy are feasible directions for managing EM.

### Electronic supplementary material

Below is the link to the electronic supplementary material.

Supplementary Fig. 1. MR leave-one-out sensitivity analysis for HDL on EM

Supplementary Fig. 2. MR leave-one-out sensitivity analysis for LDL on EM

Supplementary Fig. 3. MR leave-one-out sensitivity analysis for logTG on EM

Supplementary Fig. 4. MR leave-one-out sensitivity analysis for TC on EM

Supplementary Fig. 5. Forest map results of reverse MR analysis. (A) HDL; (B) LDL; (C)LogTG; (D) TC

Supplementary Fig. 6. The plot of reverse MR analysis by leave-one-out. (A) HDL; (B) LDL; (C)LogTG; (D) TC

Supplementary Material 7

Supplementary Material 8

## Data Availability

The GWAS summary statistics for endometriosis are available on the IEU GWAS database (https://gwas.mrcieu.ac.uk/) for FinnGen [14]. The GWAS summary statistics for blood lipid are available in The Global Lipids Genetics Consortium aggregated [12, 13].
