# Supplementary figures and images for: Bidirectional causality between the levels of blood lipids and endometriosis: a two-sample mendelian randomization study
Source: BMC Womens Health. 2024 Jul 4;24:387. doi: 10.1186/s12905-024-03213-w (PMC11223312; doi:10.1186/s12905-024-03213-w)

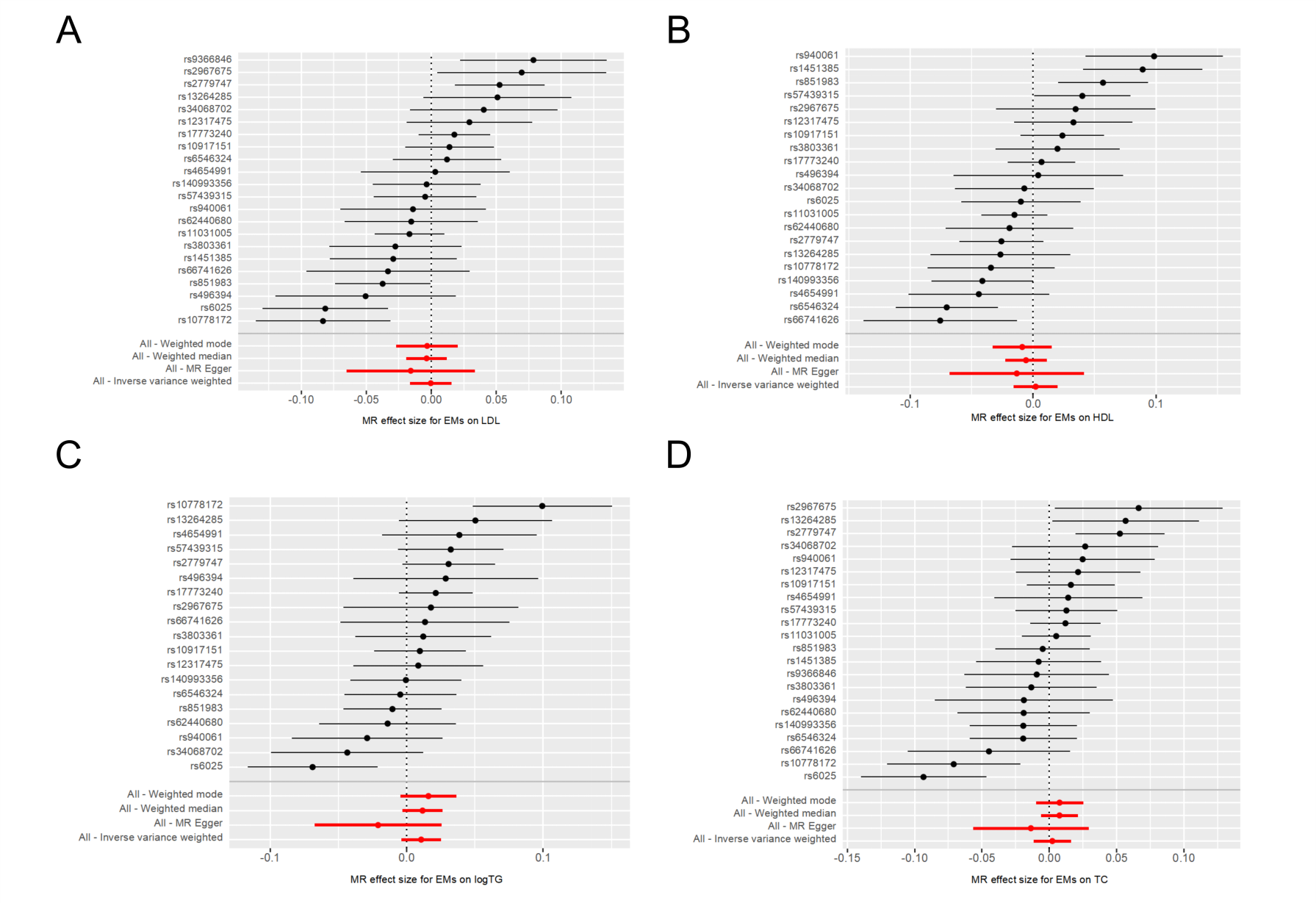

Supplement: Supplementary file 3 — Supplementary Fig. 3. MR leave-one-out sensitivity analysis for logTG on EM [file 12905_2024_3213_MOESM3_ESM.tif]

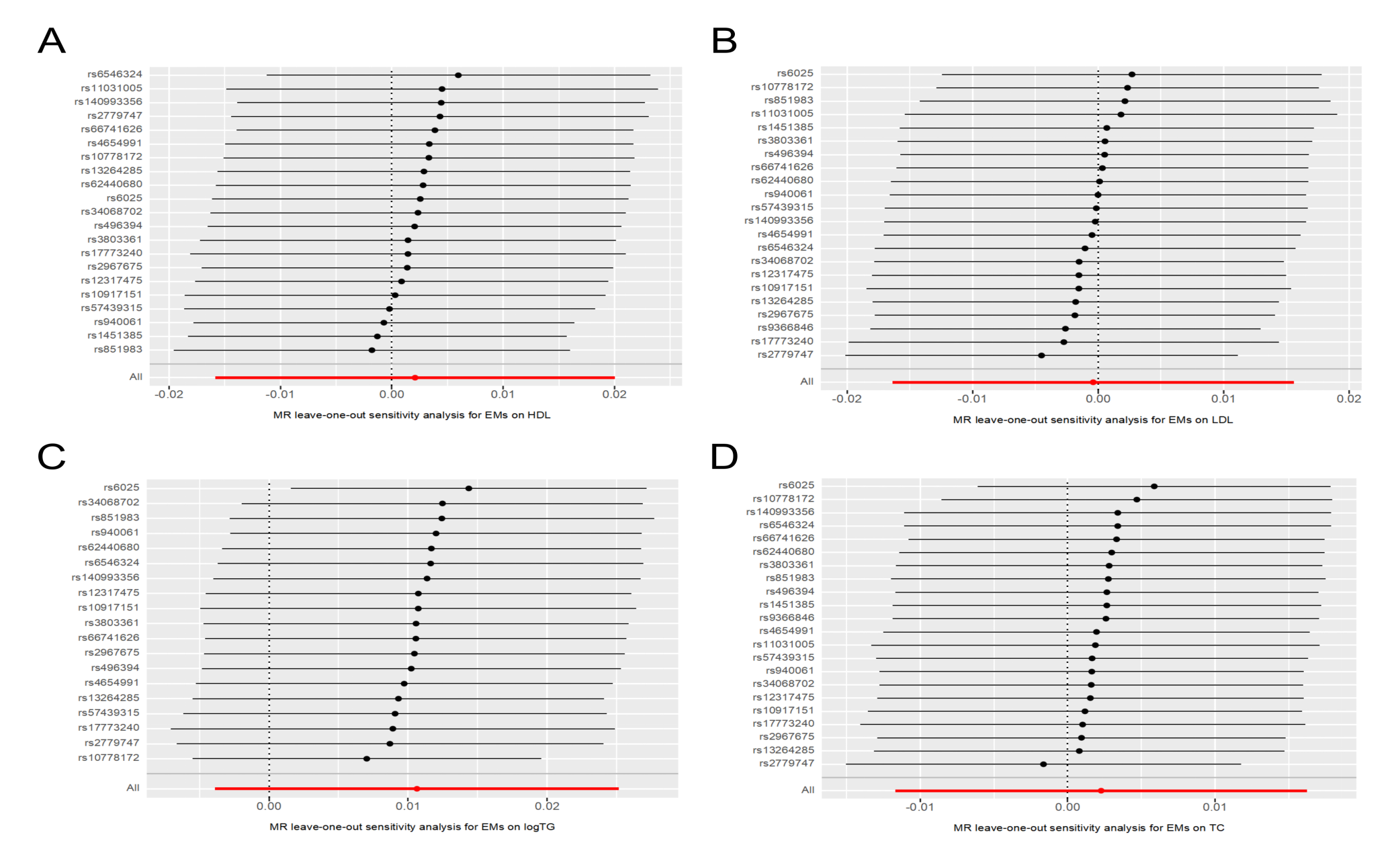

Supplement: Supplementary file 4 — Supplementary Fig. 4. MR leave-one-out sensitivity analysis for TC on EM [file 12905_2024_3213_MOESM4_ESM.tif]
