# Supplementary material for: Bidirectional causality between the levels of blood lipids and endometriosis: a two-sample mendelian randomization study
Source: BMC Womens Health. 2024 Jul 4;24:387. doi: 10.1186/s12905-024-03213-w (PMC11223312; doi:10.1186/s12905-024-03213-w)
Supplement: Supplementary file 7 — Supplementary Material 7 [file 12905_2024_3213_MOESM7_ESM.docx]

Supplementary Table 1: MR results of four blood lipids based on the location of lesions.

| Blood lipid | location of lesions | Method | SNPs | *β* | *SE* | *P* | *OR95%(CI)* |
| --- | --- | --- | --- | --- | --- | --- | --- |
| HDL | Deep endometriosis | Inverse variance weighted | 207 | 0.023 | 0.077 | 0.77 | 1.023（0.879-1.190） |
| HDL | Deep endometriosis | MR Egger | 207 | -0.014 | 0.125 | 0.91 | 0.986(0.772-1.259) |
| HDL | Deep endometriosis | Weighted median | 207 | 0.036 | 0.123 | 0.77 | 1.037(0.815-1.320) |
| HDL | Deep endometriosis | Weighted mode | 207 | 0.013 | 0.126 | 0.92 | 1.0130(0.791-1.296) |
| HDL | Endometriosis of fallopian tube | Inverse variance weighted | 206 | -0.353 | 0.281 | 0.21 | 0.703(0.405-1.218) |
| HDL | Endometriosis of fallopian tube | MR Egger | 206 | 0.281 | 0.450 | 0.53 | 1.324(0.549-3.196) |
| HDL | Endometriosis of fallopian tube | Weighted median | 206 | -0.127 | 0.450 | 0.78 | 0.880(0.365-2.127) |
| HDL | Endometriosis of fallopian tube | Weighted mode | 206 | 0.486 | 0.558 | 0.38 | 1.627(0.545-4.856) |
| HDL | Endometriosis of intestine | Inverse variance weighted | 206 | 0.277 | 0.189 | 0.14 | 1.320(0.911-1.911) |
| HDL | Endometriosis of intestine | MR Egger | 206 | 0.570 | 0.304 | 0.06 | 1.769(0.975-3.210) |
| HDL | Endometriosis of intestine | Weighted median | 206 | 0.288 | 0.295 | 0.33 | 1.334(0.748-2.379) |
| HDL | Endometriosis of intestine | Weighted mode | 206 | 0.438 | 0.310 | 0.16 | 1.549(0.844-2.843) |
| HDL | Unspecified | Inverse variance weighted | 205 | -0.122 | 0.082 | 0.14 | 0.885(0.754-1.039) |
| HDL | Unspecified | MR Egger | 205 | -0.089 | 0.132 | 0.50 | 0.914(0.706-1.184) |
| HDL | Unspecified | Weighted median | 205 | -0.134 | 0.121 | 0.27 | 0.875(0.690-1.110) |
| HDL | Unspecified | Weighted mode | 205 | -0.110 | 0.141 | 0.43 | 0.895(0.679-1.180) |
| HDL | Endometriosis of ovary | Inverse variance weighted | 205 | -0.089 | 0.058 | 0.13 | 0.915(0.816-1.025) |
| HDL | Endometriosis of ovary | MR Egger | 205 | -0.041 | 0.094 | 0.66 | 0.960(0.799-1.153) |
| HDL | Endometriosis of ovary | Weighted median | 205 | -0.049 | 0.082 | 0.55 | 0.952(0.811-1.117) |
| HDL | Endometriosis of ovary | Weighted mode | 205 | -0.043 | 0.088 | 0.63 | 0.958(0.806-1.138) |
| HDL | Endometriosis of pelvic peritoneum | Inverse variance weighted | 204 | -0.050 | 0.065 | 0.45 | 0.951(0.837-1.081) |
| HDL | Endometriosis of pelvic peritoneum | MR Egger | 204 | -0.022 | 0.106 | 0.83 | 0.978(0.795-1.203) |
| HDL | Endometriosis of pelvic peritoneum | Weighted median | 204 | 0.001 | 0.096 | 0.99 | 1.001(0.829-1.208) |
| HDL | Endometriosis of pelvic peritoneum | Weighted mode | 204 | -0.025 | 0.116 | 0.83 | 0.975(0.778-1.223) |
| HDL | Endometriosis of rectovaginal septum and vagina | Inverse variance weighted | 207 | -0.024 | 0.087 | 0.78 | 0.976(0.823-1.158) |
| HDL | Endometriosis of rectovaginal septum and vagina | MR Egger | 207 | -0.017 | 0.140 | 0.90 | 0.983(0.746-1.294) |
| HDL | Endometriosis of rectovaginal septum and vagina | Weighted median | 207 | 0.005 | 0.126 | 0.97 | 1.005(0.785-1.287) |
| HDL | Endometriosis of rectovaginal septum and vagina | Weighted mode | 207 | 0.036 | 0.126 | 0.78 | 1.036(0.810-1.325) |
| HDL | Endometriosis of uterus | Inverse variance weighted | 206 | -0.178 | 0.069 | 0.01 | 0.837(0.731-0.959) |
| HDL | Endometriosis of uterus | MR Egger | 206 | -0.184 | 0.112 | 0.10 | 0.832(0.668-1.035) |
| HDL | Endometriosis of uterus | Weighted median | 206 | -0.282 | 0.100 | 0.00 | 0.754(0.620-0.918) |
| HDL | Endometriosis of uterus | Weighted mode | 206 | -0.253 | 0.102 | 0.01 | 0.777(0.636-0.948) |
| LDL | Deep endometriosis | Inverse variance weighted | 210 | 0.081 | 0.069 | 0.23 | 1.085(0.948-1.241) |
| LDL | Deep endometriosis | MR Egger | 210 | 0.076 | 0.094 | 0.42 | 1.079(0.897-1.298) |
| LDL | Deep endometriosis | Weighted median | 210 | 0.094 | 0.106 | 0.38 | 1.098(0.892-1.353) |
| LDL | Deep endometriosis | Weighted mode | 210 | 0.059 | 0.091 | 0.52 | 1.061(0.887-1.269) |
| LDL | Endometriosis of fallopian tube | Inverse variance weighted | 210 | 0.061 | 0.237 | 0.80 | 1.062(0.667-1.691) |
| LDL | Endometriosis of fallopian tube | MR Egger | 210 | 0.280 | 0.326 | 0.39 | 1.323(0.698-2.505) |
| LDL | Endometriosis of fallopian tube | Weighted median | 210 | -0.011 | 0.399 | 0.98 | 0.989(0.453-2.161) |
| LDL | Endometriosis of fallopian tube | Weighted mode | 210 | 0.041 | 0.360 | 0.91 | 1.041(0.514-2.108) |
| LDL | Endometriosis of intestine | Inverse variance weighted | 210 | 0.233 | 0.166 | 0.16 | 1.262(0.912-1.747) |
| LDL | Endometriosis of intestine | MR Egger | 210 | 0.352 | 0.228 | 0.12 | 1.422(0.909-2.223) |
| LDL | Endometriosis of intestine | Weighted median | 210 | 0.208 | 0.264 | 0.43 | 1.231(0.734-2.065) |
| LDL | Endometriosis of intestine | Weighted mode | 210 | 0.236 | 0.230 | 0.30 | 1.267(0.808-1.986) |
| LDL | Unspecified | Inverse variance weighted | 210 | 0.092 | 0.065 | 0.16 | 1.096(0.966-1.244) |
| LDL | Unspecified | MR Egger | 210 | 0.036 | 0.089 | 0.68 | 1.037(0.871-1.234) |
| LDL | Unspecified | Weighted median | 210 | 0.010 | 0.112 | 0.93 | 1.010(0.811-1.259) |
| LDL | Unspecified | Weighted mode | 210 | 0.003 | 0.088 | 0.97 | 1.003(0.844-1.192) |
| LDL | Endometriosis of ovary | Inverse variance weighted | 210 | 0.039 | 0.055 | 0.48 | 1.040(0.933-1.160) |
| LDL | Endometriosis of ovary | MR Egger | 210 | -0.043 | 0.076 | 0.57 | 0.958(0.826-1.112) |
| LDL | Endometriosis of ovary | Weighted median | 210 | -0.016 | 0.087 | 0.85 | 0.984(0.829-1.168) |
| LDL | Endometriosis of ovary | Weighted mode | 210 | -0.023 | 0.069 | 0.74 | 0.977(0.854-1.119) |
| LDL | Endometriosis of pelvic peritoneum | Inverse variance weighted | 206 | 0.077 | 0.050 | 0.12 | 1.080(0.979-1.191) |
| LDL | Endometriosis of pelvic peritoneum | MR Egger | 206 | 0.094 | 0.068 | 0.17 | 1.099(0.961-1.256) |
| LDL | Endometriosis of pelvic peritoneum | Weighted median | 206 | 0.101 | 0.077 | 0.19 | 1.106(0.952-1.286) |
| LDL | Endometriosis of pelvic peritoneum | Weighted mode | 206 | 0.098 | 0.069 | 0.16 | 1.103(0.964-1.262) |
| LDL | Endometriosis of rectovaginal septum and vagina | Inverse variance weighted | 210 | 0.044 | 0.077 | 0.57 | 1.045(0.898-1.216) |
| LDL | Endometriosis of rectovaginal septum and vagina | MR Egger | 210 | 0.016 | 0.106 | 0.88 | 1.016(0.825-1.252) |
| LDL | Endometriosis of rectovaginal septum and vagina | Weighted median | 210 | -0.012 | 0.116 | 0.91 | 0.988(0.787-1.239) |
| LDL | Endometriosis of rectovaginal septum and vagina | Weighted mode | 210 | -0.009 | 0.099 | 0.93 | 0.991(0.816-1.203) |
| LDL | Endometriosis of uterus | Inverse variance weighted | 209 | 0.074 | 0.059 | 0.21 | 1.077(0.959-1.209) |
| LDL | Endometriosis of uterus | MR Egger | 209 | 0.042 | 0.081 | 0.61 | 1.043(0.889-1.223) |
| LDL | Endometriosis of uterus | Weighted median | 209 | 0.044 | 0.090 | 0.62 | 1.045(0.876-1.248) |
| LDL | Endometriosis of uterus | Weighted mode | 209 | 0.041 | 0.087 | 0.64 | 1.041(0.879-1.234) |
| logTG | Deep endometriosis | Inverse variance weighted | 189 | 0.007 | 0.087 | 0.93 | 1.007(0.850-1.194) |
| logTG | Deep endometriosis | MR Egger | 189 | -0.139 | 0.128 | 0.28 | 0.871(0.677-1.120) |
| logTG | Deep endometriosis | Weighted median | 189 | -0.176 | 0.124 | 0.16 | 0.838(0.658-1.069) |
| logTG | Deep endometriosis | Weighted mode | 189 | -0.153 | 0.112 | 0.17 | 0.858(0.689-1.068) |
| logTG | Endometriosis of fallopian tube | Inverse variance weighted | 189 | 0.666 | 0.294 | 0.02 | 1.946(1.093-3.464) |
| logTG | Endometriosis of fallopian tube | MR Egger | 189 | 1.021 | 0.438 | 0.02 | 2.777(1.177-6.549) |
| logTG | Endometriosis of fallopian tube | Weighted median | 189 | 0.798 | 0.443 | 0.07 | 2.220(0.931-5.295) |
| logTG | Endometriosis of fallopian tube | Weighted mode | 189 | 0.962 | 0.408 | 0.02 | 2.618(1.177-5.822) |
| logTG | Endometriosis of intestine | Inverse variance weighted | 189 | -0.387 | 0.202 | 0.06 | 0.679(0.457-1.010) |
| logTG | Endometriosis of intestine | MR Egger | 189 | -0.485 | 0.302 | 0.11 | 0.616(0.341-1.112) |
| logTG | Endometriosis of intestine | Weighted median | 189 | -0.575 | 0.321 | 0.07 | 0.563(0.300-1.057) |
| logTG | Endometriosis of intestine | Weighted mode | 189 | -0.486 | 0.305 | 0.11 | 0.615(0.338-1.119) |
| logTG | Unspecified | Inverse variance weighted | 187 | 0.093 | 0.082 | 0.26 | 1.098(0.934-1.290) |
| logTG | Unspecified | MR Egger | 187 | 0.062 | 0.123 | 0.61 | 1.064(0.837-1.353) |
| logTG | Unspecified | Weighted median | 187 | 0.063 | 0.133 | 0.64 | 1.065(0.821-1.382) |
| logTG | Unspecified | Weighted mode | 187 | 0.118 | 0.123 | 0.34 | 1.125(0.883-1.433) |
| logTG | Endometriosis of ovary | Inverse variance weighted | 189 | 0.139 | 0.057 | 0.02 | 1.149(1.027-1.286) |
| logTG | Endometriosis of ovary | MR Egger | 189 | 0.142 | 0.086 | 0.10 | 1.152(0.974-1.362) |
| logTG | Endometriosis of ovary | Weighted median | 189 | 0.187 | 0.090 | 0.04 | 1.206(1.010-1.439) |
| logTG | Endometriosis of ovary | Weighted mode | 189 | 0.140 | 0.078 | 0.08 | 1.150(0.986-1.341) |
| logTG | Endometriosis of pelvic peritoneum | Inverse variance weighted | 187 | 0.171 | 0.070 | 0.01 | 1.186(1.035-1.360) |
| logTG | Endometriosis of pelvic peritoneum | MR Egger | 187 | 0.210 | 0.103 | 0.04 | 1.234(1.008-1.512) |
| logTG | Endometriosis of pelvic peritoneum | Weighted median | 187 | 0.070 | 0.100 | 0.49 | 1.073(0.881-1.306) |
| logTG | Endometriosis of pelvic peritoneum | Weighted mode | 187 | 0.155 | 0.085 | 0.07 | 1.167(0.989-1.378) |
| logTG | Endometriosis of rectovaginal septum and vagina | Inverse variance weighted | 189 | 0.067 | 0.094 | 0.48 | 1.069(0.890-1.284) |
| logTG | Endometriosis of rectovaginal septum and vagina | MR Egger | 189 | -0.077 | 0.139 | 0.58 | 0.926(0.705-1.216) |
| logTG | Endometriosis of rectovaginal septum and vagina | Weighted median | 189 | -0.079 | 0.137 | 0.57 | 0.924(0.706-1.209) |
| logTG | Endometriosis of rectovaginal septum and vagina | Weighted mode | 189 | -0.113 | 0.115 | 0.33 | 0.894(0.713-1.119) |
| logTG | Endometriosis of uterus | Inverse variance weighted | 189 | 0.052 | 0.074 | 0.48 | 1.053(0.911-1.217) |
| logTG | Endometriosis of uterus | MR Egger | 189 | 0.131 | 0.110 | 0.23 | 1.140(0.919-1.414) |
| logTG | Endometriosis of uterus | Weighted median | 189 | 0.106 | 0.114 | 0.35 | 1.112(0.889-1.391) |
| logTG | Endometriosis of uterus | Weighted mode | 189 | 0.125 | 0.105 | 0.24 | 1.133(0.922-1.391) |
| TC | Deep endometriosis | Inverse variance weighted | 221 | 0.033 | 0.071 | 0.64 | 1.034(0.899-1.189) |
| TC | Deep endometriosis | MR Egger | 221 | 0.086 | 0.102 | 0.40 | 1.090(0.892-1.331) |
| TC | Deep endometriosis | Weighted median | 221 | -0.013 | 0.113 | 0.91 | 0.988(0.791-1.233) |
| TC | Deep endometriosis | Weighted mode | 221 | 0.027 | 0.103 | 0.79 | 1.027(0.840-1.257) |
| TC | Endometriosis of fallopian tube | Inverse variance weighted | 221 | 0.169 | 0.243 | 0.49 | 1.185(0.736-1.907) |
| TC | Endometriosis of fallopian tube | MR Egger | 221 | 0.050 | 0.348 | 0.88 | 1.052(0.532-2.079) |
| TC | Endometriosis of fallopian tube | Weighted median | 221 | -0.086 | 0.440 | 0.85 | 0.918(0.388-2.173) |
| TC | Endometriosis of fallopian tube | Weighted mode | 221 | -0.018 | 0.395 | 0.96 | 0.983(0.453-2.132) |
| TC | Endometriosis of intestine | Inverse variance weighted | 221 | 0.283 | 0.170 | 0.10 | 1.327(0.951-1.852) |
| TC | Endometriosis of intestine | MR Egger | 221 | 0.463 | 0.243 | 0.06 | 1.589(0.987-2.559) |
| TC | Endometriosis of intestine | Weighted median | 221 | 0.285 | 0.299 | 0.34 | 1.329(0.739-2.390) |
| TC | Endometriosis of intestine | Weighted mode | 221 | 0.281 | 0.271 | 0.30 | 1.324(0.778-2.252) |
| TC | Unspecified | Inverse variance weighted | 220 | 0.040 | 0.070 | 0.56 | 1.041(0.908-1.193) |
| TC | Unspecified | MR Egger | 220 | 0.079 | 0.100 | 0.43 | 1.082(0.890-1.315) |
| TC | Unspecified | Weighted median | 220 | 0.014 | 0.114 | 0.90 | 1.014(0.811-1.269) |
| TC | Unspecified | Weighted mode | 220 | 0.045 | 0.105 | 0.67 | 1.046(0.851-1.285) |
| TC | Endometriosis of ovary | Inverse variance weighted | 221 | -0.005 | 0.058 | 0.93 | 0.995(0.888-1.115) |
| TC | Endometriosis of ovary | MR Egger | 221 | 0.028 | 0.083 | 0.74 | 1.028(0.873-1.211) |
| TC | Endometriosis of ovary | Weighted median | 221 | -0.017 | 0.092 | 0.85 | 0.983(0.821-1.177) |
| TC | Endometriosis of ovary | Weighted mode | 221 | -0.011 | 0.076 | 0.88 | 0.989(0.852-1.149) |
| TC | Endometriosis of pelvic peritoneum | Inverse variance weighted | 218 | 0.060 | 0.055 | 0.27 | 1.061(0.954-1.181) |
| TC | Endometriosis of pelvic peritoneum | MR Egger | 218 | 0.168 | 0.077 | 0.03 | 1.183(1.017-1.376) |
| TC | Endometriosis of pelvic peritoneum | Weighted median | 218 | 0.151 | 0.085 | 0.07 | 1.163(0.985-1.373) |
| TC | Endometriosis of pelvic peritoneum | Weighted mode | 218 | 0.146 | 0.079 | 0.07 | 1.157(0.991-1.351) |
| TC | Endometriosis of rectovaginal septum and vagina | Inverse variance weighted | 221 | -0.006 | 0.078 | 0.94 | 0.994(0.854-1.157) |
| TC | Endometriosis of rectovaginal septum and vagina | MR Egger | 221 | 0.008 | 0.111 | 0.94 | 1.008(0.811-1.254) |
| TC | Endometriosis of rectovaginal septum and vagina | Weighted median | 221 | -0.053 | 0.124 | 0.67 | 0.949(0.743-1.211) |
| TC | Endometriosis of rectovaginal septum and vagina | Weighted mode | 221 | -0.051 | 0.111 | 0.65 | 0.951(0.764-1.182) |
| TC | Endometriosis of uterus | Inverse variance weighted | 220 | -0.008 | 0.058 | 0.89 | 0.992(0.886-1.112) |
| TC | Endometriosis of uterus | MR Egger | 220 | -0.040 | 0.083 | 0.63 | 0.960(0.816-1.131) |
| TC | Endometriosis of uterus | Weighted median | 220 | 0.026 | 0.103 | 0.80 | 1.027(0.838-1.257) |
| TC | Endometriosis of uterus | Weighted mode | 220 | -0.018 | 0.094 | 0.85 | 0.982(0.817-1.181) |
