# Supplementary material for: Bidirectional causality between the levels of blood lipids and endometriosis: a two-sample mendelian randomization study
Source: BMC Womens Health. 2024 Jul 4;24:387. doi: 10.1186/s12905-024-03213-w (PMC11223312; doi:10.1186/s12905-024-03213-w)
Supplement: Supplementary file 8 — Supplementary Material 8 [file 12905_2024_3213_MOESM8_ESM.docx]

Supplementary Table 2: Egger intercept test results based on the location of lesions.

| Blood lipid | location of lesions | Intercept | *SE* | *P* |
| --- | --- | --- | --- | --- |
| HDL | Deep endometriosis | 0.001 | 0.004 | 0.705 |
|  | Endometriosis of fallopian tube | -0.025 | 0.014 | 0.074 |
|  | Endometriosis of intestine | -0.012 | 0.009 | 0.220 |
|  | Unspecified | -0.001 | 0.004 | 0.751 |
|  | Endometriosis of ovary | -0.002 | 0.003 | 0.512 |
|  | Endometriosis of pelvic peritoneum | -0.001 | 0.003 | 0.742 |
|  | Endometriosis of rectovaginal septum and vagina | 0.000 | 0.004 | 0.951 |
|  | Endometriosis of uterus | 0.000 | 0.003 | 0.944 |
| LDL | Deep endometriosis | 0.000 | 0.004 | 0.933 |
|  | Endometriosis of fallopian tube | -0.012 | 0.012 | 0.328 |
|  | Endometriosis of intestine | -0.007 | 0.009 | 0.447 |
|  | Unspecified | 0.003 | 0.003 | 0.364 |
|  | Endometriosis of ovary | 0.005 | 0.003 | 0.116 |
|  | Endometriosis of pelvic peritoneum | -0.001 | 0.003 | 0.713 |
|  | Endometriosis of rectovaginal septum and vagina | 0.002 | 0.004 | 0.704 |
|  | Endometriosis of uterus | 0.002 | 0.003 | 0.563 |
| logTG | Deep endometriosis | 0.006 | 0.004 | 0.127 |
|  | Endometriosis of fallopian tube | -0.015 | 0.014 | 0.274 |
|  | Endometriosis of intestine | 0.004 | 0.010 | 0.660 |
|  | Unspecified | 0.001 | 0.004 | 0.732 |
|  | Endometriosis of ovary | 0.000 | 0.003 | 0.968 |
|  | Endometriosis of pelvic peritoneum | -0.002 | 0.003 | 0.605 |
|  | Endometriosis of rectovaginal septum and vagina | 0.006 | 0.004 | 0.165 |
|  | Endometriosis of uterus | -0.003 | 0.003 | 0.331 |
| TC | Deep endometriosis | -0.003 | 0.004 | 0.470 |
|  | Endometriosis of fallopian tube | 0.006 | 0.012 | 0.633 |
|  | Endometriosis of intestine | -0.009 | 0.009 | 0.301 |
|  | Unspecified | -0.002 | 0.003 | 0.591 |
|  | Endometriosis of ovary | -0.002 | 0.003 | 0.579 |
|  | Endometriosis of pelvic peritoneum | -0.005 | 0.003 | 0.050 |
|  | Endometriosis of rectovaginal septum and vagina | -0.001 | 0.004 | 0.857 |
|  | Endometriosis of uterus | 0.002 | 0.003 | 0.587 |
